# Venlafaxine Attenuates Heat Hyperalgesia Independent of Adenosine or Opioid System in a Rat Model of Peripheral Neuropathy

**Published:** 2015

**Authors:** Alireza Abed, Valiollah Hajhashemi, Hamid Reza Banafshe, Mohsen Minaiyan, Azam Mesdaghinia

**Affiliations:** a *Department of Pharmacology, School of Medicine, Kashan University of Medical Sciences, Kashan, Iran.*; b *Department of Pharmacology and Toxicology and Isfahan Pharmaceutical Research Center, School of Pharmacy and Pharmaceutical Sciences, Isfahan University of Medical Sciences, Isfahan, Iran.*; c *Physiology Research Center, Kashan University of Medical Sciences, Kashan, Iran.*; d *Department of Addiction Studies, School of Medicine, Kashan University of Medical Sciences, Kashan, Iran.*

**Keywords:** Venlafaxine, Neuropathic pain, Naloxone, Caffeine, Chronic constriction injury

## Abstract

Primarily opioidergic and adenosine mechanisms are considered to be involved in the antinociceptive effects of antidepressants. This study was designed to determine the efficacy of acute venlafaxine administration in alleviating symptoms of neuropathic pain and the role of endogenous adenosine and opioid systems in this effect of venlafaxine. We have evaluated the effect of caffeine, a non-selective adenosine A_1_ and A_2_ receptor antagonist and naloxone as an antagonist of opioid receptors on the antinociceptive effects of venlafaxine. Chronic constriction injury of the sciatic nerve resulted in thermal hyperalgesia, mechanical and cold allodynia in the rats. Animals were received on the 7^th^ day after surgery, when the model had been fully established, venlafaxine (20 and 40 mg/Kg *i.p*.), or venlafaxine (40 mg/Kg) in combination with caffeine (5 mg/Kg *i.p*.) or naloxone (1 mg/Kg *s.c*.). Rats were tested for thermal reaction latencies, mechanical and cold allodynia 45 min after drug injection. Acute venlafaxine (40 mg/Kg *i.p*.) administration consistently decreased the thermal hyperalgesia and this effect was not blocked by concomitant caffeine or naloxone administration. There was no effect by either drug or the drug combination on the tactile and cold allodynia. The results of this study indicate that venlafaxine (40 mg/Kg *i.p*.) is effective in alleviating thermal hyperalgesia and this effect is independent through manipulation of adenosine or opioid system. This observation demonstrates that venlafaxine, which is a mixed inhibitor of norepinephrine and serotonin reuptake, differs from the other antidepressants in the mechanism of its antinociception action.

## Introduction

The ability of antidepressants, which block the reuptake of both norepinephrine and serotonin to relieve neuropathic pains, is well documented (1). Venlafaxine belongs to newer antidepressant drugs with fewer side effects compared to tricyclic antidepressants (TCA) (2). The pain alleviating properties of venlafaxine in different pain models such as neurogenic pain, diabetic neuropathy and fibromyalgia have been demonstrated (3-5). It has been shown that venlafaxine prevents and relives oxaliplatin-induced acute neurotoxicity (6). In addition, venlafaxine does not induce the usual TCA side-effects caused by their anticholinergic, anti-histaminic and alpha-1 adrenergic antagonistic properties (7). Thus, this drug could be a novel and promising pharmacological approach in peripheral neuropathic pain treatment.

The mechanisms involving the analgesic effects of venlafaxine are still poorly understood. Early research determined that venlafaxine at the supraspinal and spinal levels increased the amount of noradrenalin and serotonin in the descending inhibitory pathways (8). However NMDA receptor blockade, sodium channel blockade and activation of μ-and δ-opioid receptors are proposed as possible mechanisms which may be involved in its analgesic effects (9, 10).

Some studies have suggested that analgesic effects of venlafaxine mediate through endogenous adenosine systems (11). This possibility is interesting since adenosine has been shown to have antinociceptive properties and endogenous levels of adenosine are reduced in the plasma and cerebral spinal fluid of patients with neuropathic pain (12-17). Several studies have also shown an opioidergic involvement in the antinociceptive effect of antidepressants, often demonstrated by the inhibitory effect of naloxone, an antagonist of opioidergic receptors, on their antinociception actions (18, 19).

In the present study, we have investigated whether acute venlafaxine administration was effective in alleviating symptoms of neuropathic pain and whether an adenosine or opioid-related mechanisms contribute to the antinociceptive effects of acute venlafaxine administration in a rat model of neuropathic pain. In this study we used caffeine as a non-selective adenosine receptors antagonist which is being equally effective at both adenosine A_1_ and A_2_ receptors (20) and naloxone as an opioidergic receptors antagonist.

## Experimental

*Animals and housing conditions*

The experiments were performed on male Sprague–Dawley rats (200–250 g). They were housed four per cage, in a room under controlled temperature (23±2 °C), humidity (50%) and lighting (12/12 h light/dark cycle), with food and water available ad libitum. All experiments were approved by the ethical committee of Isfahan University of Medical sciences and followed the European Commission Directive (86/609/EEC) for animal experiments.

Procedures involving animals and their care were conducted in conformity with NIH guidelines for the care and use of laboratory animals.

*Neuropathic pain model*

The rats were anesthetized with ketamine (50 mg/Kg *i.p*.) and xylazine (10 mg/Kg *i.p.*).The common sciatic nerve was exposed and dissected from surrounding connective tissue near the trocanter, just distal to the branching point of the posterior biceps semitendinosus nerve. Four ligatures (4.0 chromic Gut) were tied loosely around the nerve with a 1-1.5 mm interval between ligatures so that the circulation through the superficial epineuria vasculature was not totally interrupted. Sham-operated rats had the same surgery, the left sciatic nerve was exposed but no ligation was made. The rats were housed individually in cages after the surgery (21).

*Behavioral tests of neuropathic pain*

Hyperalgesia to noxious thermal stimulus and allodynia to mechanical stimuli were determined as behavioral score of neuropathic pain by using the radiant heat plantar and von Frey test, respectively. These tests were performed during the day portion of the circadian cycle (09:00–16:00 h). After cage exploration and major grooming activities ceased, we made the behavioral tests. The behavioral scores of neuropathic pain were determined 1 day before the surgery as the baseline value and also 45 min after the injections on the 7^th^ day after the surgery (22).

*Thermal hyperalgesia (plantar test)*

Paw withdrawal latency in response to radiant heat was measured by using plantar test apparatus (UgoBasile, Varese, Italy). Rats were placed within a Plexi glass enclosure (but not restrained) on a transparent glass floor. An infrared beam that constitutes the heat source was moved beneath the mid-plantar surface of the hind paw. Thermal withdrawal latency was defined as the latency (seconds) between the heat stimulus onset and paw withdrawal using a feedback-controlled shut-down unit. A cut-off time of 22 s was used to avoid tissue damage. Each paw was tested three times alternatively at minimum intervals of 5 min between stimulation to avoid sensitization of the hind paw. Mean latency of the withdrawal response for ipsilateral (operated) and contralateral (non-operated) paws were calculated separately (23).

*Mechanical allodynia (von Frey filament stimulation)*

To examine mechanical allodynia, withdrawal threshold to mechanical stimuli was measured using von Frey filaments (steeling, Wood Dale, IL, USA) in the following order: 0.6, 1.0, 1.4, 2.0, 4.0, 6.0, 8.0, 10.0, 15.0, 26.0 and 60 g.

Rats were placed on a mesh (0.8×0.8 cm cell) floor, covered by an inverted transparent plastic box (18×18×25 cm) and allowed to adapt for approximately 15 min, or until exploratory behavior ceased. A series of von Frey filament stimuli were delivered in an ascending order of forces to the central region of the plantar surface of the hind paw. The stimulation was applied three times consecutively, pushing down on the hind paw until the rat withdrew its paw or the fiber bowed. Lifting of the paw due to normal locomotors behavior was ignored. The smallest filament size which evoked at least 3 withdrawal responses during 5 consecutive applications was considered as withdrawal threshold. Each filament was applied for approximately 1 s and the inter stimulus intervals were about 5 s (24).

*Cold allodynia (acetone test)*

Cold allodynia was performed with using the acetone spray test (evaporation-evoked cooling) as described previously. Rats were placed on a wire mesh floor; acetone bubbles formed at the end of a tube connected to a syringe were applied 5 times (at 5 min intervals) to the plantar surface of the hind paw. The frequency of paw withdrawal was expressed as a percentage (the number of paw withdrawals/number of trials×100) (24).

*Treatments*

Animals were randomly assigned into following 8 groups (*n*=6). Control group: in this group common sciatic nerve was exposed then four ligatures (4.0 chromic Gut) were tied loosely around the nerve. Rats were treated with normal saline (5 mL/Kg *i.p*.). 

 Sham group: Sham-operated rats had the same surgery, the left sciatic nerve was exposed but no nerve ligations were made and rats were treated with normal saline (5 mL/Kg *i.p*.). 

Treatment groups: CCI rats who were treated with venlafaxine (20 and 40 mg/Kg *i.p*.) on the 7^th^ day after surgery.

 Caffeine group: CCI animals who were received venlafaxine (40 mg/Kg *i.p*.) concurrent caffeine (5 mg/Kg *i.p*.) on the 7^th^ day after surgery.

Naloxone group: CCI animals who were received venlafaxine (40 mg/Kg *i.p*.) concurrent naloxone (1 mg/Kg *s.c*.) on the 7^th^ day after surgery.

Rats were injected with the vehicle (saline solution 0.9%) and the baseline paw withdrawal thresholds were measured. Then behavioral tests were measured at 45 minutes after acute injection of venlafaxine (20 and 40 mg/Kg *i.p*.). Tests were done on the 7^th^ day after surgery. Caffeine (5 mg/Kg *i.p*.) and naloxone (1 mg/Kg *s.c*.) were administered separately in combination with venlafaxine (40 mg/Kg *i.p*.), 15 minutes before venlafaxine administration, and behavioral tests were done 45 minutes after venlafaxine injection. 

*Statistical analysis*

 Data were compared by one-way analysis of variance (ANOVA) followed by Fisher LSD post-hoc test for multiple comparisons. 

## Results

*Behavioral tests of neuropathic pain*

The rats did not show any sign of autotomy after the sciatic nerve ligation. Paw gesture of the ipsilateral paw was slightly altered; but this did not interfere with the normal activity of the rats. There was no evidence for the occurrence of contralateral hyperalgesia and allodynia in all tested groups.

*Thermal hyperalgesia*

Sciatic nerve ligation decreased paw withdrawal latency to the thermal stimulus in ipsilateral paw, but sham operation did not produce any significant change in the paw withdrawal latency.

**Figure 1:**
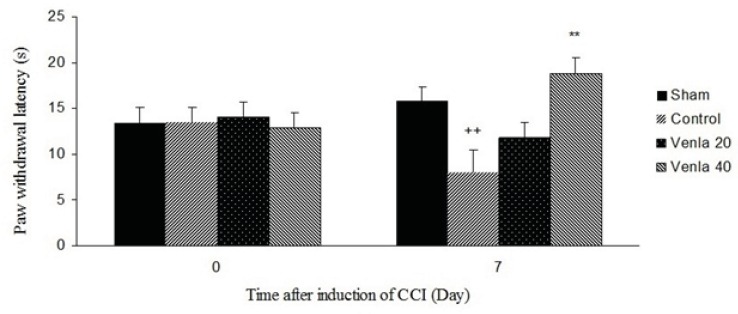
The effects of acute treatment with venlafaxine (20 and 40 mg/Kg *i.p*.) on heat hyperalgesia. Sham group (n = 6): rats who were subjected to sham treatments (Saline solution 5 mL/Kg) for chronic constrictive nerve injury (CCI), Control group (n = 6): chronic constrictive nerve injury of the right sciatic nerve was performed, Venla 20: CCI animals who were treated with venlafaxine (20 mg/Kg *i.p*.), Venla 40: CCI animals who were treated with venlafaxine (40 mg/Kg *i.p*.).

Acute administration of venlafaxine (40 mg/Kg *i.p*.) 45 minute before the plantar test on the 7^th^ day after surgery blocked thermal hyperalgesia in ipsilateral paw (P<0.01) (Figure 1). 

The results are expressed as Mean ± S.E.M., n=6 in all groups.**P<0.01 versus control group, ++P<0.01 versus sham group.

*Mechanical allodynia*

**Figure 2:**
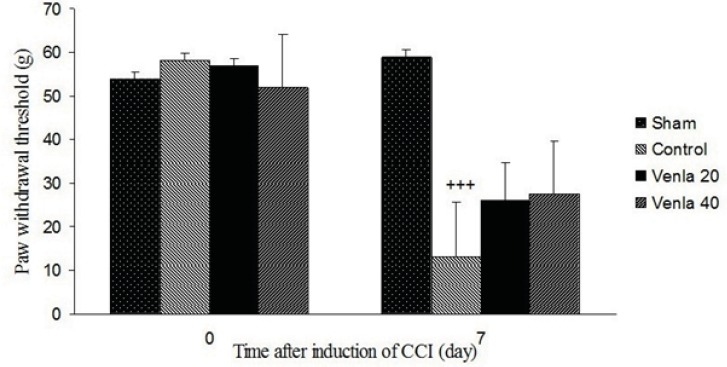
The effects of acute venlafaxine (20 and 40 mg/Kg *i.p*.) administration on the mechanical allodynia. Sham group (n = 6): rats who were subjected to sham treatments (Saline solution 5 mL/Kg) for chronic constrictive nerve injury (CCI), Control group (n = 6): chronic constrictive nerve injury of the right sciatic nerve was performed, Venla 20: CCI animals who were treated with venlafaxine (20 mg/Kg *i.p*.), Venla 40: CCI animals who were treated with venlafaxine (40 mg/Kg *i.p*.).

Sciatic nerve ligation led to a significant decrease of the withdrawal threshold of ipsilateral paw in comparison with sham operated group (P<0.001). Acute treatment with venlafaxine (20 and 40 mg/Kg *i.p*.) couldn’t significantly modify withdrawal threshold of ipsilateral paw (Figure 2).

The results are expressed as Mean ± S.E.M., n=6 in all groups. +++P<0.01 versus sham group.

*The effects of caffeine on theantinociceptive effects of venlafaxine*

**Figure 4:**
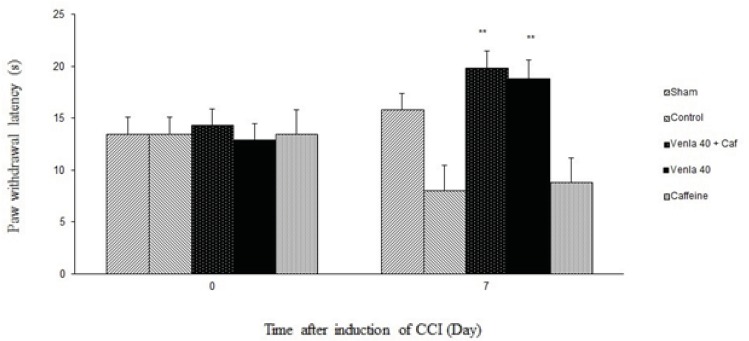
The effect of caffeine (5 mg/Kg *i.p*.) on antihyperagesic effect of acute treatment with venlafaxine (40 mg/Kg *i.p*.).

Caffeine (5 mg/Kg *i.p*.) didn’t significantly prevent the antihyperalgesic effect of acute venlafaxine injection (40 mg/Kg *i.p*.) in the radiant heat plantar test (Figure 4).

Sham group (n = 6): rats who were subjected to sham treatments (Saline solution 5 mL/Kg) for chronic constrictive nerve injury (CCI), Control group (n = 6): chronic constrictive nerve injury of the right sciatic nerve was performed, Venla 40: CCI animals who were treated with venlafaxine (40 mg/Kg *i.p*.), Caffeine: CCI animals who were treated with caffeine (5 mg/Kg *i.p*.), Venla 40+ Caf: CCI animals who were received venlafaxine (40 mg/Kg *i.p*.) concurrent caffeine (5 mg/Kg *i.p*.).The results are expressed as Mean ± S.E.M., n=6 in all groups**P<0.01 versus control group.

*The effects of naloxone on the antinociceptive effects of venlafaxine*

**Figure 5:**
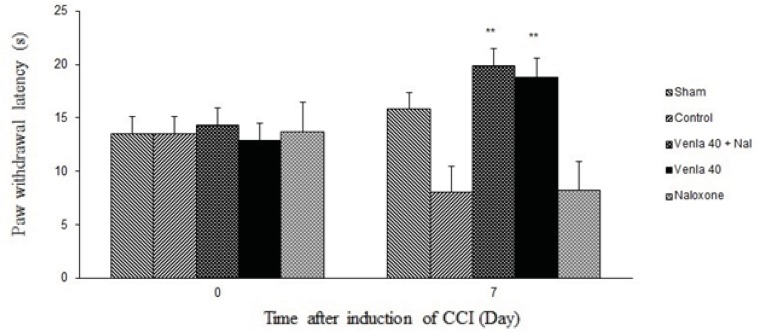
The effect of naloxone (1 mg/Kg *s.c*.) on the antihyperagesic effect of acute treatment with venlafaxine (40 mg/Kg *i.p*.).

Figure 5 shows the effect of naloxone (1 mg/Kg *s.c*.) on the antihyperalgesic effect of acute venlafaxine injection (40 mg/Kg *i.p*.). Naloxone could not change antinociceptive effects of venlafaxine in the radiant heat plantar test.

Sham group (n = 6): rats who were subjected to sham treatments (Saline solution 5 mL/Kg) for chronic constrictive nerve injury (CCI), Control group (n = 6): chronic constrictive nerve injury of the right sciatic nerve was performed, Venla 40: CCI animals who were treated with venlafaxine (40 mg/Kg *i.p*.), Naloxone: CCI animals who were treated with naloxone (1 mg/Kg s.c.),Venla 40+ Nal: CCI animals who were received venlafaxine (40 mg/Kg *i.p*.) concurrent naloxone (1 mg/Kg *s.c*.).

The results are expressed as Mean ± S.E.M., n=6 in all groups**P<0.01 versus control group.

## Discussion

The present study demonstrates that acute venlafaxine (40 mg/Kg *i.p*.) administration on the 7^th^ day after surgery, once the neuropathic pain had been fully established, inhibits heat hyperalgesia expression in an opioidergic or adenosine independent manner.

Recently anti-inflammatory and analgesic effects of some antidepressant have been demonstrated (25-27).

Venlafaxine is a serotonin/ norepinephrine reuptake inhibitor (28). But in comparison with other dual re-uptake inhibitors such as duloxetine has weak affinity for serotonin and norepinephrine transporters. Thus, in rats, at low doses (<10 mg/Kg *i.p*.) acts as an SSRI and only begin to exhibit dual re-uptake properties at doses >30 mg/Kg *i.p*. mainly due to increasing concentrations of the R-O-desmethylvenlafaxine, an active metabolite of venlafaxine (29). 

Considering The results of this study and other studies, it can be proposed that the maximal anti-nociceptive effect of venlafaxine obtained when it was administered at high doses (>30 mg/Kg) in the rats (30, 31). 

It has been demonstrated that the opioidergic system is involved in the effect of tricyclic antidepressants (TCAs) (32). The opioidergic system also proposed to be involved in the antihyperalgesic effect of venlafaxine (30). However, according to our results the inability of naloxone to reduce the antihyperalgesic effect of venlafaxine seems to exclude this possibility. These results also demonstrate that venlafaxine, which is a mixed inhibitor of norepinephrine and serotonin reuptake, differs from the other antidepressants in the mechanism of its antinociception action. 

Schreiber et al have reported that the antinociceptive effect of venlafaxine in the hot plate model of thermal pain in mice is mediated through opioid and adrenergic mechanisms (30). They conclude that venlafaxine reduced noradrenergic responsiveness which may induce an indirect activation of the opioid system. On the other hand Marchand and his colleagues reported that the antihyperalgesic effect of venlafaxine in diabetic rats does not involve the opioid system (33). It is well known that different animal models of neuropathic pain have different pathogenesis. In this study we have used chronic constriction injury of sciatic nerve as an animal model of peripheral neuropathic pain. This model is based on a unilateral loose ligation of the sciatic nerve, which is one of the most frequently used models for the study of neuropathic pain and its treatment (34, 35). This model also shows many of the pathophysiological properties of chronic neuropathic pain in human subjects, such as allodynia and hyperalgesia (36). Our results showed that venlafaxine antinociception effects in the peripheral neuropathy are mediated independent of the opioid system.

It is widely documented that the adenosine system is involved in the effect of tricyclic antidepressants, possibly in relation to their monoaminergic action (37). The adenosine system could also be involved in the antihyperalgesic effect of venlafaxine (11). However, according to our results the inability of the caffeine to reduce the antihyperalgesic effect of venlafaxine in the peripheral neuropathy seems to rule out this possibility. However it has been shown that caffeine significantly antagonize the analgesic effect of venlafaxine in the mice model of hot plate test (11). This observation also demonstrates that venlafaxine, which is a mixed inhibitor of norepinephrine and serotonin reuptake, produce its analgesic effects differs from TCAs such as amitriptyline (38).

## Conclusion

The present study indicates a clear antihyperalgesic effect of venlafaxine in peripheral neuropathy. This effect is not inhibited by naloxone or caffeine which, interestingly, distinguishes venlafaxine from TCAs. These results suggest a potential use of venlafaxine in management of neuropathic pain. 

## References

[1] SindrupS, JensenT (1999). Efficacy of pharmacological treatments of neuropathic pain: an update and effect related to mechanism of drug action. Pain.

[2] Qiu B, Qiao J, Yong J (2014). Meta-analysis of Selective Serotonin Reuptake Inhibitors (SSRIs) compared to tricyclic antidepressants (TCAs) in the efficacy and safety of anti-depression Therapy in Parkinson᾽s Disease (PD) Patients. Iran. J. Pharm. Res.

[3] Sumpton JE, Moulin DE (2001). Treatment of neuropathic pain with venlafaxine. Ann. Pharmacother.

[4] Cegielska-Perun K, Bujalska-Zadrożny M, Tatarkiewicz J, Gąsińska E, Elżbieta H, Nowak M (2013). Venlafaxine and neuropathic pain. Pharmacol.

[5] DwightMM, Arnold LM, O’Brien H, Metzger R, Morris-Park E, Keck PE (1998). An open clinical trial of venlafaxine treatment of fibromyalgia. Psychosomatics.

[6] Durand JP, Deplanque G, Montheil V, Gornet JM, Scotte F, Mir O (2012). Efficacy of venlafaxine for the prevention and relief of oxaliplatin-induced acute neurotoxicity: results of EFFOX, a randomized, double-blind, placebo-controlled phase III trial. Ann. Oncol.

[7] Ellingrod VL, Perry PJ (1994). Venlafaxine: a heterocyclic antidepressant. Am. J. Hosp. Pharm.

[8] Lee YC, Chen PP (2010). A review of SSRIs and SNRIs in neuropathic pain. Expert. Opin. Pharmacother.

[9] Wrzosek A, Obara I, Wordliczek J, Przewlocka B (2009). Efficacy of tramadol in combination with doxepin or venlafaxine in inhibition of nociceptive process in the rat model of neuropathicpain: an isobolographic analysis. J. Physiol. Pharmacol.

[10] Hajhashemi V, Sadeghi H, Minaiyan M, Movahedian A, Talebi A (2011). Effect of Fluvoxamine on carrageenan-induced paw edema in rats evaluation of the action sites. Iran. J. Pharm. Res.

[11] Yaba G, Sezer Z, Tekol Y (2006). Interaction between venlafaxine and caffeine on antinociceptionin mice. Pharmazia.

[12] SawynokJ (1998). Adenosine receptor activation and nociception. Eur. J. Pharmacol.

[13] Sawynok J, Reid A, Esser MJ (1999). Peripheral antinociceptive action of amitriptyline in the rat formalin test: involvement of adenosine. Pain.

[14] Belfrage M, Sollevi A, Segerdahl M, Sjolund K-F, Hansson P (1995). Systemic adenosine infusion alleviates spontaneous and stimulus evoked pain in patients with peripheral neuropathic pain. Anesth. Analg.

[15] Rane K, Segerdahl M, Goiny M, Sollevi A (1998). Intrathecal adenosine administration. A phase 1 clinical safety study in healthy volunteers, with additional evaluation of its influence on sensory thresholds and experimental pain. Anesthesiol.

[16] Sollevi A, Belfrage M, Lundeberg T, Segerdahl M, Hansson P (1995). Systemic adenosine infusion: a new treatment modality to alleviate neuropathic pain. Pain.

[17] Guieu R, Peragut JC, Roussel P, Hassani H, Sampieri F, Bechis G, Gola R, Rochat H (1996). Adenosine and neuropathic pain. Pain.

[18] Ardid D, Guilbaud G (1992). Antinociceptive effects of acute and chronic injections of tricyclic antidepressant drugs in a new model of mononeuropathy in rats. Pain.

[19] Gray AM, Spencer PS, Sewell RD (1998). The involvement of the opioidergic system in the antinociceptive mechanism of action of antidepressant compounds. Br. J. Pharmacol.

[20] Fredholm BR (1995). Adenosine, adenosine receptors and the actions of caffeine. Pharmacol. Toxicol.

[21] Zimmermann M (2001). Pathobiology of neuropathic pain. Eur. J. Pharmacol.

[22] Amin B, Hajhashemi V, Hosseinzadeh H, Abnous Kh (2012). Antinociceptive evaluation of ceftriaxone and minocycline alone and in combination in a neuropathic pain model in rat. Neurosci.

[23] Banafshe H, HamidiGA, Noureddini M, Mirhashemi M, Mokhtaria M, Shoferpour M (2014). Effect of curcumin on diabetic peripheral neuropathic pain: Possible involvement of opioid system. Eur. J. Pharmacol.

[24] Banafshe HR, Mesdaghinia A, Arani MN, Ramezani MH, Heydari A, Hamidi GA (2012). Lithium attenuates pain-related behavior in a rat model of neuropathic pain: possible involvement of opioid system. Pharmacol. Biochem. Behav.

[25] Hajhashemi V, Banafshe HR, Minaiyan M, Mesdaghinia A, Abed A (2014). Antinociceptive effects of venlafaxine in a rat model of peripheral neuropathy: Role of alpha2-adrenergic receptors. Eur. J. Pharmacol.

[26] Hajhashemi V, Sadeghi H, Minaiyan M, Movahedian A, Talebi A (2010). Central andperipheral anti-inflammatory effects of maprotiline on carrageenan-induced paw edema in rats. Inflamm. Res.

[27] Sadeghi H, Hajhashemi V, Minaiyan M, Movahedian A, Talebi A (2011). A study on the mechanisms involving the anti-inflammatory effect of amitriptyline in carrageenan-induced paw edema in rats. Eur. J. Pharmacol.

[28] Baldessarini RJ, JG Hardman, LE Limbird, A Goodman Gilman (2001). Drugs for the treatment of psychiatric disorders. Goodman & Gilman’s The Pharmacological Basis of Therapeutics.

[29] Pedersen LH, Nielsen AN, Munro GB (2005). Anti-nociception is selectively enhanced by parallel inhibition of multiple subtypes of monoamine transporters in rat models of persistent and neuropathic pain. Psychopharmacol.

[30] Schreiber S, Backer M, Pick C (1999). The antinociceptive effect of venlafaxine in mice is mediated through opioid and adrenergic mechanisms. Neurosci. Lett.

[31] Koch S, Hemrick-Luecke SK, Thompson LK, Evans DC, Threlkeld PG, Nelson DL (2003). Comparison of effects of dual transporter inhibitors on monoamine transporters and extracellular levels in rats. Neuropharmacol.

[32] Eschalier A, Ardid D, Dubray C, Sawynok, C Alan (1999). Tricyclic and other antidepressantsas analgesics. Novel Aspects of Pain Management: Opioids and Beyond.

[33] Marchand F, Alloui A, Chapuy E, Hernandez A, Pelissier T, Ardid D, Eschalier A (2003). The antihyperalgesic effect of venlafaxine in diabetic rats does not involve the opioid system. Neurosci. Lett.

[34] Verdi J, Jafari-Sabet M, Mokhtari R, Mesdaghinia A, Banafshe HR (2013). The effect of progesterone on expression and development of neuropathic pain in a rat model of peripheral neuropathy. Eur. J. Pharmacol.

[35] Hamidi GA, Ramezani MH, Arani MN, Talaei SA, Mesdaghinia A, Banafshe HR (2012). Ethosuximide reduces allodynia and hyperalgesia and potentiates morphine effects in the chronic constriction injury model of neuropathic pain. Eur. J. Pharmacol.

[36] Choi Y, Yoon YW, Na HS, Kim SH, Chung JM (1994). Behavioral signs of ongoing pain and cold allodynia in a rat model of neuropathic pain. Pain.

[37] Esser MJ, Sawynok J (2000). Caffeine blockade of the thermal antihyperalgesic effect of acute amitriptyline in a rat model of neuropathic pain. Eur. J. Pharmacol.

[38] Esser MJ, Chase T, Allen GV, Sawynok J (2001). Chronic administration of amitriptyline and caffeine in a rat model of neuropathic pain: multiple interactions. Eur. J. Pharmacol.

